# OLDER AGE AND DEPRESSION AS THE RISK FACTORS FOR HYPERTENSION AMONG NORTH KOREAN DEFECTORS

**DOI:** 10.1093/geroni/igae098.3020

**Published:** 2024-12-31

**Authors:** Sang Hui Chu, Oksim Kim, Hokon Kim

**Affiliations:** Yonsei University College of Nursing, Mo-Im Kim Nursing Research Institute, Seoul, Republic of Korea; Yonsei University College of Nursing, Seoul, Republic of Korea; Yonsei University College of Nursing, Seoul, Republic of Korea

## Abstract

Observations indicate a low obesity prevalence among North Korean Defectors (NKRs), who fled to South Korea, attributed to undernourishment prior to their arrival. With an aging NKR demographic, there’s growing concern over chronic disease management, including hypertension. Previous studies reported a high or comparable prevalence of elevated blood pressure among NKRs, yet the influence of mental health on hypertension has been underexplored. Given the high rates of trauma-related conditions and depression within this group, these factors could significantly elevate hypertension risk. Understanding hypertension risk factors across different age groups could inform tailored management strategies for older NKRs. This study assessed the impact of mental health on hypertension among 449 NKRs categorized into three age groups: younger (< 30 years; n=141), middle-aged (40-59 years; n=243), and older (≥ 60 years; n=65). The assessment of Post-Traumatic Stress Disorder (PTSD)/complex PTSD (CPTSD) employed the International Trauma Questionnaire (ITQ), covering six domains: re-experiencing, avoidance, sense of threat, affective dysregulation, negative self-concept, and disturbance in relationships. Hypertension and depression were identified through self-reported diagnoses. Logistic regression analysis revealed that, in addition to older age, depression was significantly associated with hypertension, but not PTSD/CPTSD, after controlling for gender, living alone, and residency period in South Korea. These findings suggest the necessity of prioritizing mental health improvement, especially depression, in hypertension management among NKRs. This work was supported by the Basic Science Research Program through the National Research Foundation (NRF) of Korea funded by the Ministry of Education (2019R1I1A2A01058746).

